# Atrophic hypothyroidism associated with thyrotropin receptor antibodies

**DOI:** 10.31744/einstein_journal/2025RC1400

**Published:** 2025-08-12

**Authors:** Marcos Tadashi Kakitani Toyoshima, Camila Regina Pereira Batista de Macedo, Isabelle Pinheiro Amaro de Magalhaes, Janaina Almeida de Souza, Giulianno Dias da Rocha, Suemi Marui

**Affiliations:** 1 Universidade de São Paulo Faculdade de Medicina Hospital das Clínicas São Paulo SP Brazil Endocrinology Service, Hospital das Clínicas, Faculdade de Medicina, Universidade de São Paulo, São Paulo, SP, Brazil.; 2 Faculdade de Medicina de Jundiaí Jundiaí SP Brazil Faculdade de Medicina de Jundiaí, Jundiaí, SP, Brazil.

**Keywords:** Hypothyroidism, Hashimoto disease, Thyroiditis, Thyroiditis, autoimmune, Thyrotropin, Long-acting thyroid stimulator, Pregnancy, Receptors, thyrotropin, Autoantibodies

## Abstract

Hypothyroidism is most frequently caused by Hashimoto's thyroiditis. While thyrotropin receptor antibodies are well-known in Graves’ disease-induced hyperthyroidism, their role in hypothyroidism is emerging. We report the case of a 37-year-old woman with facial and periorbital edema, weight gain, and hoarseness suggestive of hypothyroidism. Elevated thyroid stimulating hormone (TSH; 131mIU/L) and decreased free thyroxine (0.2ng/dL, 2.6pmol/L) levels confirmed this diagnosis. Laboratory findings showed dyslipidemia, impaired renal function, elevated creatine phosphokinase levels, and anemia. Despite negative results for thyroid peroxidase (TPO-Ab) and thyroglobulin (TgAb) antibodies, positive thyrotropin receptor antibodies (11IU/L) suggested an autoimmune etiology. Thyroid ultrasound confirmed atrophic thyroiditis. Levothyroxine treatment improved the symptoms and laboratory values. Two months after diagnosis, the patient became pregnant. Anemia relapsed during pregnancy, thyrotropin receptor antibodies levels normalized, and postpartum follow-up revealed stable thyroid function without affecting the newborn thyroid function. This case underscores the diagnostic challenges of negative TPOAb and TgAb hypothyroidism, which is often misdiagnosed as Hashimoto's thyroiditis, and highlights the need for functional discrimination in thyrotropin receptor antibodies assays. Thyrotropin receptor antibodies measurements can help in the differential diagnosis of rapidly evolving hypothyroidism in women with negative TPO-Ab and thyrotropin receptor antibodies levels, especially in women of childbearing age. Further research is essential to understand thyrotropin-binding inhibitory immunoglobulin-mediated hypothyroidism and to develop targeted therapies.

## INTRODUCTION

Hypothyroidism is a prevalent endocrine disorder resulting from insufficient thyroid hormone production. Hashimoto's thyroiditis is the leading cause of hypothyroidism in iodine-sufficient regions.^( 1 - 3 )^ While thyrotropin receptor antibodies (TRAb) are well established in hyperthyroidism due to Graves’ disease, their significance in hypothyroidism is gaining recognition.^( 1 )^ TRAb can be categorized into three types: thyroid-stimulating immunoglobulins/antibodies (TSI), which mimic the action of thyrotropin or thyroid-stimulating hormone (TSH) and lead to Graves’ disease; thyrotropin-binding inhibitory immunoglobulins/antibodies (TBII), which block the action of TSH; or functionally neutral antibodies, which may induce apoptosis but have a minimal role in impairing thyroid function.^( 1 , 4 - 6 )^ This case report aims to shed light on the diagnostic and therapeutic challenges posed by negative thyroid peroxidase (TPO-Ab) and thyroglobulin antibodies (TgAb), and TRAb-positive or TBII-positive hypothyroidism.

## CASE REPORT

A 37-year-old woman presented with a 2-month history of facial and periorbital edema, weight gain, and hoarseness suggestive of hypothyroidism. The patient had no known personal or family history of thyroid disease or significant medical comorbidities.

### Diagnostic assessment

**Figure 1:**
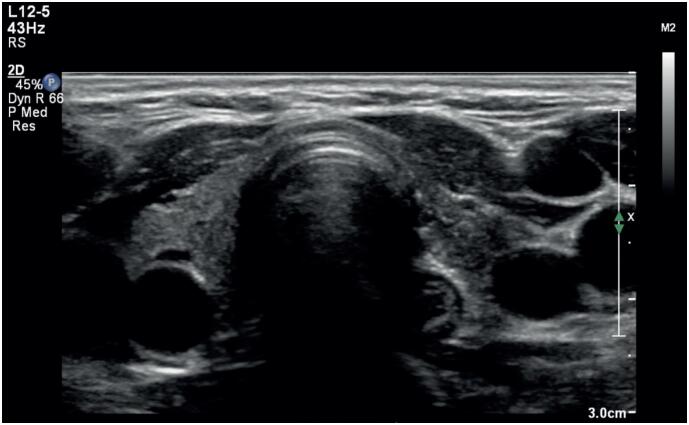
Ultrasonography of the thyroid gland reveals a marked reduction in size, indicating an overall atrophic appearance (total thyroid volume, 4.2cm^3^; normal range, 6-15cm^3^) in a 37-year-old woman with atrophic hypothyroidism associated with elevated thyrotropin receptor antibodies

Upon physical examination, the patient was slightly pale and had facial edema. There was no goiter on thyroid examination. Laboratory investigations revealed very high thyroid stimulating hormone (TSH) levels (131mIU/L; reference normal range, 0.4-4.5mIU/L) and decreased free thyroxine (fT4) levels, consistent with primary hypothyroidism. Additionally, the patient had significant dyslipidemia, impaired renal function, elevated creatine phosphokinase, and normochromic normocytic anemia. A previous evaluation (8 months prior) revealed normal thyroid and renal functions and lipid profiles. Given the absence of TPO-Ab and TgAb, and the rapid evolution of hypothyroidism, a TRAb test was requested, which showed a positive result ( Table 1 ) using a competitive electrochemiluminescence immunoassay (Roche Diagnostics Ltd., Basel, Switzerland; normal value <1IU/L), indicating an autoimmune etiology. A thyroid ultrasound was performed to confirm the suspicion of atrophic hypothyroidism, revealing heterogeneous and hypoechoic parenchyma with a small total volume of 4.2cm^3^ (normal range, 6-15cm^3^) ( Figure 1 ).

**Table 1:** Evolution of clinical and laboratory data of a 37-year-old woman with atrophic hypothyroidism associated with elevated thyrotropin receptor antibodies

Variable	Normal range	8 months prior to the diagnosis	(On initial evaluation) Jan 16, 2023	1 month after the diagnosis	GI 4 weeks 4 days (Pregnancy diagnosis)	GI 17 weeks 3 days	GI 27 weeks 3 days	GI 32 weeks 3 days	3-month postpartum	6-month postpartum
Levothyroxine daily dose				100mcg	112mcg	125mcg	137mcg	125mcg	100mcg	100mcg
TSH	0.4-4.5mIU/L	1.5mIU/L	131.0mIU/L	26.0mIU/L	10.2mIU/L	1.5mIU/L	0.9mIU/L	0.6mIU/L	0.9mIU/L	0.8mIU/L
fT4	0.9-1.7ng/dL (11.6-21.9pmol/L)		0.2ng/dL (2.6pmol/L)	1.3ng/dL (16.7pmol/L)	1.2ng/dL (15.5pmol/L)	1.3ng/dL (16.7pmol/L)	1.0ng/dL (12.9pmol/L)	0.9ng/dL (11.6pmol/L)	1.0ng/dL (12.9pmol/L)	1.1ng/dL (14.2pmol/L)
TRAb	<1.0IU/L			11.0IU/L	7.0IU/L	3.0IU/L	1.0IU/L	1.0IU/L	<1.0IU/L	1.0IU/L
TPOAb	<34IU/mL (<34kIU/L)			<34IU/mL (<34kIU/L)			<34IU/mL (<34kIU/L)			34IU/mL (<34kIU/L
TgAb	<115IU/mL (<115kIU/L)			36IU/mL (36kIU/L)			14IU/mL (14kIU/L)			20IU/mL (20kIU/L)
Creatinine	0.60-1.10mg/dL (53.0-97.2mcmol/L)	0.93mg/dL (82.2mcmol/L)	1.40mg/dL (123.8mcmol/L)		0.94mg/dL (83.1mcmol/L)				0.99mg/dL (87.5mcmol/L)	0.87mg/dL (76.9mcmol/L)
Hemoglobin	11.7-14.9g/dL (117-149g/L)		10.6g/dL (106g/L)		10.9g/dL (109g/L)	11.2g/dL (112g/L)	10.8g/dL (108g/L)	11.8g/dL (118g/L)	12.6g/dL (126g/L)	13.3g/dL (133g/L)
Hematocrit	35.1-44.1% (0.35-0.44 L/L)		33.5% (0.34 L/L)		34.3% (0.34 L/L)	33.8% (0.34 L/L)	33.8% (0.34 L/L)	36.0% (0.36 L/L)	39.0% (0.39 L/L)	40.6% (0.41 L/L)
Total cholesterol	<190mg/dL (<4.9mmol/L)	167mg/dL (4.3mmol/L)	274mg/dL (7.1mmol/L)		149mg/dL (3.9mmol/L)					154mg/dL (4.0mmol/L)
LDL- cholesterol	<130mg/dL (<3.4mmol/L)	98mg/dL (2.5mmol/L)	187mg/dL (4.8mmol/L)		86mg/dL (2.2mmol/L)					93mg/dL (2.4mmol/L)
HDL- cholesterol	>50mg/dL (>1.3mmol/L)	56mg/dL (1.5mmol/L)	72mg/dL (1.9mmol/L)		50mg/dL (1.3mmol/L)					47mg/dL (1.2mmol/L)
Triglyceride	<150mg/dL (<1.7mmol/L)	51mg/dL (0.6mmol/L)	54mg/dL (0.6mmol/L)		45mg/dL (0.5mmol/L)					58mg/dL (0.7mmol/L)
CK	26-140IU/L		457IU/L		98IU/L					
hCG	<2IU/L				7135IU/L					

CK: creatine kinase; fT4: free thyroxine; GI: gestational age; hCG: human chorionic gonadotropin; HDL: high-density lipoprotein; LDL: low-density lipoprotein; TgAb: thyroglobulin antibody; TPOAb: Anti-thyroid peroxidase antibody; TRAb: anti-TSH receptor antibody; TSH: thyroid stimulating hormone.

### Treatment

The initial dose of oral levothyroxine was 1.6mcg/kg/day. Statin therapy was not considered for the treatment of dyslipidemia.

### Outcome and follow-up

The evolution of laboratory test results during the follow-up period is shown in table 1 . One month after starting levothyroxine, there was an improvement in clinical symptoms, normalization of fT4 levels, lipid profile (cholesterol, 149mg/dL, 3.9mmol/L; low-density lipoprotein cholesterol (LDL-c), 86mg/dL, 2.2mmol/L), renal function (glomerular filtration rate (GFR), 79mL/min/1.73m^2^), and creatinine kinase (CK; 98IU/L), in addition to an improvement in anemia and a drop in TRAb (7IU/L), with adjustments in levothyroxine doses. TRAb measurements were repeated in another laboratory that routinely used the TSI Immulite chemiluminometric assay (Siemens Healthcare, Llanberis, UK) to identify only TSI. In this assay, the sample was negative (normal value <0.55IU/L). The QUEST-TSI bioassay for the TSI functional activity yielded negative results.

Two months later, the patient discovered that she had become pregnant. The levothyroxine dose was adjusted throughout pregnancy, which was uneventful, except for maternal anemia requiring parenteral iron replacement. From week 23 of pregnancy, the TRAb level remained within the reference limit for positivity (1.0IU/L). At week 40 of pregnancy, the baby was born by forceps delivery due to rotation dystocia, with a birth weight of 6.15 pounds (2790g) and a height of 19.7 inches (50cm). The neonatal heel prick test indicated TSH = 4.97mIU/L (immunofluorimetric method; reference value, <13.2mIU/L). There were no complications during the 6-month postpartum follow-up, with a stable maternal levothyroxine dose and negative TRAb values from week 12 after birth.

Signed informed consent has been obtained directly from the patient. This study was approved by the Research Ethics Committee of *Hospital das Clínicas da Faculdade de Medicina da Universidade de São Paulo* (CAAE: 87073725.2.0000.0068; #7.498.038).

## DISCUSSION

The patient's clinical course, marked by significant edema, hoarseness, and rapid weight gain, coupled with markedly elevated TSH and low fT4 levels, was suggestive of severe hypothyroidism. This case highlights the diagnostic challenges of negative TPO-Ab and TgAb hypothyroidism, which are often misdiagnosed as Hashimoto's thyroiditis. The presence of TRAb was critical in identifying the autoimmune etiology of her condition. TRAb poses diagnostic challenges, given the limited availability of assays to discriminate between functional antibody types. This case highlights the limitations of conventional thyroid function tests in capturing the intricacies of autoimmune-mediated atrophic hypothyroidism.

Atrophic hypothyroidism associated with TBII is an uncommon clinical entity that is often misdiagnosed as Hashimoto's thyroiditis; however, this condition may also be associated with Hashimoto's thyroiditis.^( 7 )^ Although both Hashimoto's and atrophic thyroiditis can cause hypothyroidism, their pathophysiological mechanisms differ. In Hashimoto's thyroiditis, cell-mediated immune responses involving Th1 and CD4+ T helper lymphocytes are predominant. In contrast, atrophic thyroiditis is characterized by the activation of Th2 CD4+ T helper lymphocytes, which assist B lymphocytes in producing TRAb. This process ultimately leads to thyroid atrophy and replacement of the normal parenchyma with fibrous tissue.^( 2 , 8 )^

The two types of laboratory assays most commercially used in clinical practice are assays that measure only TRAb without functional discrimination, which is the most widely used, and bioassays that measure the functional activity of TRAb.^( 1 )^ Bioassays for detecting TSI and TBII activity have been improved and replaced by assays using cell cultures that express the TSH receptor more stably.^( 4 , 9 - 11 )^ In the present case, measuring the TBII activity was impossible. Therefore, tests to detect TSI and its stimulatory activity were performed, both of which yielded negative results, reinforcing the hypothesis of atrophic hypothyroidism caused by TBII.

It is controversial whether routine TRAb measurements should be performed in all cases of hypothyroidism in clinical practice, as the treatment strategy does not change with the results.^( 1 )^ Some authors have suggested that TRAb measurement may be useful for the differential diagnosis in patients with hypothyroidism and negative TPOAb and TgAb levels, especially in women of childbearing age diagnosed with hypothyroidism at the time of pregnancy planning or in the early stages of pregnancy.^( 9 , 12 )^ The incidence of neonatal hypothyroidism due to TBII in North America is uncommon, accounting for around 2% of cases.^( 13 )^ Identifying TRAb could assist in prenatal management, given that the transplacental passage of TBII could lead to transient hypothyroidism in neonates.^( 4 , 11 , 14 )^

Commercially available assays and advances in our understanding of TBII may pave the way for new diagnostic and therapeutic strategies for autoimmune thyroid diseases. Future research should focus on elucidating the precise molecular pathways involved in TBII-mediated suppression of thyroid function and exploring their potential as therapeutic targets to avoid atrophic hypothyroidism.

## CONCLUSION

TRAb-associated atrophic hypothyroidism is part of the differential diagnosis of rapidly progressive hypothyroidism. This diagnosis is especially important in women of childbearing age because maternal TBII can cross the placenta, potencially leading to fetal or neonatal hypothyroidism. There is a lack of commercially available assays for the detection of TBII, which is important for its identification.

DATA AVAILABILITY STATEMENT

Original data generated and analyzed during this study are included in this published article.
